# Body mass index and reported contraceptive use prior to the index pregnancy among Chinese women with unintended pregnancy: a multicenter cross-sectional analysis of a prospective cohort

**DOI:** 10.1186/s12889-026-28462-2

**Published:** 2026-07-10

**Authors:** Lin Shen, Jie Li, Kaiyan Pei, Xue Jiang, Xiaomei Jiang, Lina Zhang, Zhonglan Xu, Xiuqing Zhang, Lixia Chen, Fengyan Ren, Xiuling Guo, Xiaowan Luo, Qiwei Qi, Jun Ge

**Affiliations:** 1 National Research Institute for Family Planning, Chinese Academy of Medical Sciences & Peking UnionMedical College, No. 12, Dahuisi Road, Haidian District, Beijing, 100081 China; 2 Graduate School, Chinese Academy of Medical Sciences and Peking Union Medical College, Beijing, 100006 China; 3 Department of Obstetrics & Gynecology, Peking University First Hospital, Beijing, 100034 China; 4 Changzhou Maternal and Child Health Hospital, Changzhou, 213000 China; 5 Yangzhou Maternal and Child Health Hospital, Yangzhou, 225000 China; 6 Taiyuan Maternal and Child Health Hospital, Taiyuan, 030012 China; 7 Zhangjiakou Maternal and Child Health Hospital, Zhangjiakou, 075000 China; 8 Daqing People’s Hospital, Daqing, 163000 China; 9 Zaozhuang Maternal and Child Health Hospital, Zaozhuang, 277100 China; 10 Boai Hospital of Zhongshan, Zhongshan, 528437 China; 11 Qingdao Maternal and Child Health Hospital, Qingdao, 266011 China; 12 Tanggu Maternal and Child Health Hospital, Tanggu, 300451 China

**Keywords:** Body mass index, Contraceptive use, Unintended pregnancy, Chinese populations

## Abstract

**Background:**

Unintended pregnancy remains a major public health challenge globally and in China. While body mass index (BMI) is associated with adverse obstetric and metabolic outcomes, its relationship with contraceptive use among women with unintended pregnancy remains poorly characterized in large Chinese populations. This study investigated the cross-sectional association between BMI and reported contraceptive use prior to index pregnancy, and explored its clinical and public health implications.

**Methods:**

This cross-sectional analysis utilized baseline data from a multicenter prospective cohort study conducted at 10 hospitals across 8 Chinese provinces and municipalities (May 2019–February 2022). A total of 8,125 women with unintended pregnancy were included. Multivariable logistic regression models were applied with BMI as both continuous and categorical variables (underweight, normal weight [reference], overweight, obese). Among contraceptive users, we further examined the association between BMI and the use of highly effective (HEC) versus less effective (LEC) contraception. Sensitivity analyses using random forest imputation and complete-case analysis were performed.

**Results:**

After full adjustment, each 1-unit increase in BMI was associated with 2% lower odds of reported contraceptive use (OR = 0.98, 95% CI: 0.96–0.99; *P* = 0.003). Women with overweight (OR = 0.85, 95% CI: 0.74–0.98; *P* = 0.024) and obesity (OR = 0.75, 95% CI: 0.57–0.98; *P* = 0.035) had significantly lower odds of contraceptive use compared to women with normal weight, whereas no significant association was observed for women with underweight. Among contraceptive users, BMI showed no significant association with the use of highly effective contraception (HEC) versus less effective contraception (LEC), regardless of whether BMI was modeled as a continuous or categorical variable. These findings remained robust in sensitivity analyses.

**Conclusions:**

Overweight and obesity are independently associated with lower odds of contraceptive use among Chinese women with unintended pregnancy. These findings highlight the need for targeted contraceptive counseling for women with elevated BMI to reduce the burden of unintended pregnancy.

**Supplementary Information:**

The online version contains supplementary material available at https://doi.org/10.1186/s12889-026-28462-2.

## Introduction

Unintended pregnancy is a significant global public health issue [1]. According to World Health Organization statistics, between 2015 and 2019, there were 121 million unintended pregnancies worldwide each year, with approximately 61% of these pregnancies terminated by induced abortion [2]. Despite the establishment of a comprehensive family planning and reproductive health service system in China, approximately 30% of pregnancies are unintended [3, 4]. The resultant induced abortions not only directly compromise women’s physical and psychological well-being, but also pose risks of long-term reproductive complications and impose a substantial socioeconomic burden.

In recent decades, the correlation of body mass index (BMI) with unintended pregnancy risk has been inconsistent across studies. While several investigations have revealed that elevated BMI is correlated with an increased risk for unintended pregnancy and lower contraceptive use [5–7], others have reported no significant differences in contraceptive use patterns across weight categories [8, 9]. Notably, the majority of existing evidence has been generated in Western populations, leaving a significant knowledge gap regarding this association among Chinese reproductive-age women. Furthermore, few large-scale investigations have specifically explored the correlation of BMI with reported contraceptive use prior to the index unintended pregnancy, and even fewer have distinguished between overweight and obesity or investigated the correlation between BMI and contraceptive method use stratified by effectiveness.

Therefore, this cross-sectional analysis sought to systematically examine the association between BMI and contraceptive behaviors among women with unintended pregnancy, using baseline data from a multicenter prospective cohort study conducted at 10 hospital centers in China. We hypothesize that being overweight or obese is independently associated with lower contraceptive use and altered contraceptive method use. The findings from this large real-world study may help fill the current research gap and provide robust evidence for identifying high-risk subgroups and optimizing targeted contraceptive counseling.

## Methods

### Study design and population

**Fig. 1:**
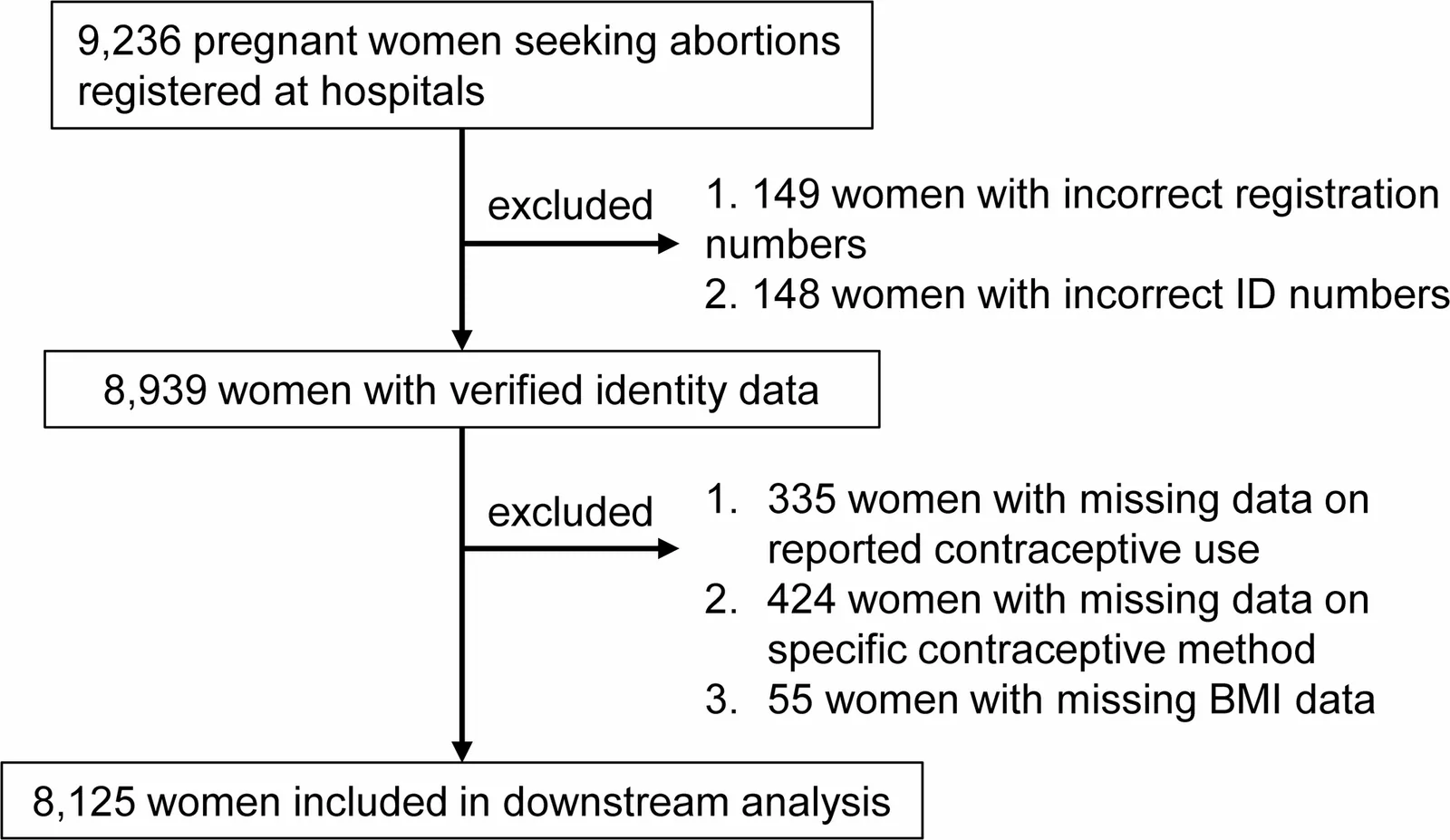
Selection process of the study population

This cross-sectional study analyzed baseline data from a large-scale prospective cohort study recruiting women with unintended pregnancy at 10 hospital centers in 8 Chinese provinces and municipalities between May 2019 and February 2022 [10]. A total of 9,236 women seeking induced abortion were initially enrolled in the cohort. We conducted a two-step data cleaning process to ensure data quality. First, we conducted identity verification: 149 records were excluded due to incorrect registration numbers, and 148 records were removed for invalid ID numbers, resulting in 8,939 women with confirmed valid identity information. Second, we excluded participants with missing data on primary exposure or outcome variables: 335 women had no data on reported contraceptive use, 424 women lacked information on the specific type of contraceptive method used, and 55 women had missing BMI data. After all exclusions, 8,125 women with complete data on all key variables were ultimately included. The participant recruitment and exclusion procedure is presented in the flow diagram (Fig. 1).

### Data collection

The following variables were included in the analysis: (1) Demographic information: BMI (kg/m^2^), hospital centers (1–10), age (years), marital status (married or others), education level (primary school education or below, education prior to bachelor`s degree, bachelor`s degree or higher), occupation (government-affiliated institutions and public sector [GIPS] or non-GIPS), monthly household income; (2) Menstrual information: age at menarche (years), menstrual cycle length (days), menstrual regularity (yes/no), menstrual duration (days), menstrual flow (estimated by number of sanitary pads used: light, < 10 pads; moderate, 20–30 pads; heavy, > 30 pads), dysmenorrhea (yes/no); (3) Obstetric and gynecologic history: history of vaginal deliveries (yes/no), history of caesarean sections (yes/no), history of spontaneous abortions (yes/no), history of induced abortions (yes/no), history of ectopic pregnancies (yes/no), history of molar pregnancies (yes/no); (4) Lifestyle and behavioral factors: smoking status (yes/no), alcohol consumption (yes/no), frequency of sexual intercourse (< 1 time/week, 1–2 times/week, ≥ 3 times/week); (5) Offspring sex composition: having at least one son (yes/no), having at least one daughter (yes/no); (6) Contraceptive outcomes: reported contraceptive use prior to the index pregnancy (yes/no, primary outcome) and contraceptive method category (secondary outcome). Contraceptive methods were categorized into two groups based on the World Health Organization (WHO) standard effectiveness classification: highly effective contraception (HEC) and less effective contraception (LEC) [11]. HEC encompassed WHO Categories 1 and 2, including subcutaneous implants, intrauterine devices, long-acting injectable contraceptives, and combined oral contraceptives. LEC encompassed WHO Category 3, including topical contraceptives (suppositories, films, gels), calendar rhythm method, withdrawal, and male condoms. The distribution of missing values for the above variables is shown in Supplementary Table 1, with the highest missing percentage observed for history of molar pregnancy (11.5%) and the lowest for smoking status (0.02%). Missing data were imputed using multiple chained equations with 10 imputed datasets and 20 iterations per imputation (R Package mice, version 3.18.0).

### Confounder selection and causal diagram construction

To minimize confounding bias and avoid inappropriate adjustment for intermediate or post-outcome variables, we employed a directed acyclic graph (DAG) approach to guide variable selection for all regression models [12, 13].

The DAG was constructed a priori using the dagitty (version 0.3-4) [14] and ggdag (version 0.2.13) [15] packages in R, based on a comprehensive review of published epidemiological evidence and established theoretical frameworks describing the relationships between BMI, contraceptive behaviors, and related factors. The DAG explicitly defined BMI as the primary exposure and contraceptive use as the primary outcome. All variables were classified into three mutually exclusive causal categories based on their causal relationships with the exposure and outcome: (1) Confounders: Variables associated with both BMI and contraceptive use that do not lie on the causal pathway between them; (2) Mediators: Variables that lie on the causal pathway from BMI to contraceptive use; (3) Other variables: Variables associated with only the exposure, only the outcome, or neither. Using the “adjustmentSets” function, we identified the minimal sufficient adjustment set required to block all backdoor paths from BMI to contraceptive use. This set included: age, marital status, education level, monthly household income, occupation, hospital center, age at menarche, history of vaginal deliveries, history of cesarean sections, smoking status, and alcohol consumption (Supplementary Fig. 1).

### Statistical analyses

BMI was categorized into four groups based on the standards established by the Working Group on Obesity in China [16] : underweight (BMI < 18.5 kg/m²), normal weight (18.5 kg/m² ≤ BMI < 24.0 kg/m²), overweight (24.0 kg/m² ≤ BMI < 28.0 kg/m²), and obese (BMI ≥ 28.0 kg/m²). Baseline characteristics of the study population were stratified by BMI category and are presented in Table 1. In descriptive statistics, continuous variables were expressed as median (interquartile range [IQR]) and evaluated comparing the Kruskal-Wallis rank sum test, while categorical variables were expressed as counts (percentages) and evaluated comparing using the chi-square test. All statistical analyses were performed using R software (version 4.4.0), with a two-sided *P*-value < 0.05 considered statistically significant.

**Table 1:** Baseline characteristics of women with unintended pregnancies

	Overall	Normal weight	Underweight	Overweight	Obese	*P*-value
*N*	8125	5641	1114	1111	259	
Hospital center						< 0.001
1	1696 (20.9%)	1112 (19.7%)	239 (21.5%)	280 (25.2%)	65 (25.1%)	
2	534 (6.6%)	363 (6.4%)	76 (6.8%)	82 (7.4%)	13 (5.0%)	
3	1872 (23.0%)	1373 (24.3%)	226 (20.3%)	224 (20.2%)	49 (18.9%)	
4	957 (11.8%)	653 (11.6%)	147 (13.2%)	132 (11.9%)	25 (9.7%)	
5	1377 (16.9%)	901 (16.0%)	200 (18.0%)	219 (19.7%)	57 (22.0%)	
6	555 (6.8%)	357 (6.3%)	77 (6.9%)	91 (8.2%)	30 (11.6%)	
7	448 (5.5%)	381 (6.8%)	28 (2.5%)	35 (3.2%)	4 (1.5%)	
8	72 (0.9%)	44 (0.8%)	14 (1.3%)	11 (1.0%)	3 (1.2%)	
9	331 (4.1%)	222 (3.9%)	77 (6.9%)	27 (2.4%)	5 (1.9%)	
10	283 (3.5%)	235 (4.2%)	30 (2.7%)	10 (0.9%)	8 (3.1%)	
Age, years	29 (25,32)	29 (25,32)	26 (23,30)	30 (26,33)	30 (26.5,33)	< 0.001
Marital status						< 0.001
Married	5837 (71.8%)	4107 (72.8%)	632 (56.7%)	884 (79.6%)	214 (82.6%)	
Others	2288 (28.2%)	1534 (27.2%)	482 (43.3%)	227 (20.4%)	45 (17.4%)	
Education level						< 0.001
Primary school education or below	919 (11.3%)	603 (10.7%)	96 (8.6%)	165 (14.9%)	55 (21.2%)	
Education prior to bachelor’s degree	4050 (49.8%)	2811 (49.8%)	566 (50.8%)	549 (49.4%)	124 (47.9%)	
Bachelor’s degree or higher	3156 (38.8%)	2227 (39.5%)	452 (40.6%)	397 (35.7%)	80 (30.9%)	
Occupation						0.027
GIPS	3827 (47.1%)	2697 (47.8%)	531 (47.7%)	495 (44.6%)	104 (40.2%)	
non-GIPS	4298 (52.9%)	2944 (52.2%)	583 (52.3%)	616 (55.4%)	155 (59.8%)	
Monthly household income						0.162
<= 4,999 RMB	3507 (43.2%)	2424 (43.0%)	487 (43.7%)	474 (42.7%)	122 (47.1%)	
5,000 ~ 9,999 RMB	2880 (35.4%)	1988 (35.2%)	421 (37.8%)	388 (34.9%)	83 (32.0%)	
>=10,000 RMB	1738 (21.4%)	1229 (21.8%)	206 (18.5%)	249 (22.4%)	54 (20.8%)	
Age at menarche, years	14 (13,14)	14 (13,14)	14 (13,15)	14 (13,14)	14 (13,14)	0.002
Menstrual cycle length, days	30 (28,30)	30 (28,30)	30 (28,30)	30 (28,30)	30 (28,30)	0.239
Menstrual regularity						< 0.001
No	688 (8.5%)	435 (7.7%)	99 (8.9%)	117 (10.5%)	37 (14.3%)	
Yes	7437 (91.5%)	5206 (92.3%)	1015 (91.1%)	994 (89.5%)	222 (85.7%)	
Menstrual duration, days	5 (5,7)	5 (5,7)	6 (5,7)	5 (5,7)	5 (5,7)	< 0.001
Menstrual flow						< 0.001
Light	1404 (17.3%)	989 (17.5%)	185 (16.6%)	199 (17.9%)	31 (12.0%)	
Moderate	6371 (78.4%)	4434 (78.6%)	887 (79.6%)	840 (75.6%)	210 (81.1%)	
Heavy	350 (4.3%)	218 (3.9%)	42 (3.8%)	72 (6.5%)	18 (6.9%)	
Dysmenorrhea						< 0.001
No pain	4513 (55.5%)	3129 (55.5%)	548 (49.2%)	681 (61.3%)	155 (59.8%)	
Pain	3612 (44.5%)	2512 (44.5%)	566 (50.8%)	430 (38.7%)	104 (40.2%)	
History of vaginal deliveries						< 0.001
No	5162 (63.5%)	3582 (63.5%)	769 (69.0%)	643 (57.9%)	168 (64.9%)	
Yes	2963 (36.5%)	2059 (36.5%)	345 (31.0%)	468 (42.1%)	91 (35.1%)	
History of cesarean sections						< 0.001
No	5936 (73.1%)	4117 (73.0%)	921 (82.7%)	746 (67.1%)	152 (58.7%)	
Yes	2189 (26.9%)	1524 (27.0%)	193 (17.3%)	365 (32.9%)	107 (41.3%)	
History of spontaneous abortions						0.005
No	7539 (92.8%)	5243 (92.9%)	1052 (94.4%)	1010 (90.9%)	234 (90.3%)	
Yes	586 (7.2%)	398 (7.1%)	62 (5.6%)	101 (9.1%)	25 (9.7%)	
History of induced abortions						< 0.001
No	4372 (53.8%)	3070 (54.4%)	637 (57.2%)	552 (49.7%)	113 (43.6%)	
Yes	3753 (46.2%)	2571 (45.6%)	477 (42.8%)	559 (50.3%)	146 (56.4%)	
History of ectopic pregnancies						0.166
No	7953 (97.9%)	5520 (97.9%)	1099 (98.7%)	1081 (97.3%)	253 (97.7%)	
Yes	172 (2.1%)	121 (2.1%)	15 (1.3%)	30 (2.7%)	6 (2.3%)	
History of molar pregnancies						0.644
No	8073 (99.4%)	5603 (99.3%)	1110 (99.6%)	1103 (99.3%)	257 (99.2%)	
Yes	52 (0.6%)	38 (0.7%)	4 (0.4%)	8 (0.7%)	2 (0.8%)	
Smoking status						< 0.001
Non-smoker	7902 (97.3%)	5510 (97.7%)	1058 (95.0%)	1079 (97.1%)	255 (98.5%)	
Smoker	223 (2.7%)	131 (2.3%)	56 (5.0%)	32 (2.9%)	4 (1.5%)	
Alcohol consumption						0.027
Non-drinker	7346 (90.4%)	5088 (90.2%)	993 (89.1%)	1030 (92.7%)	235 (90.7%)	
Drinker	779 (9.6%)	553 (9.8%)	121 (10.9%)	81 (7.3%)	24 (9.3%)	
Frequency of sexual intercourse						0.013
< 1 time per week	4053 (49.9%)	2855 (50.6%)	524 (47.0%)	540 (48.6%)	134 (51.7%)	
1–2 times per week	3635 (44.7%)	2477 (43.9%)	515 (46.2%)	529 (47.6%)	114 (44.0%)	
>=3 times per week	437 (5.4%)	309 (5.5%)	75 (6.7%)	42 (3.8%)	11 (4.2%)	
Having at least one son						< 0.001
No	5271 (64.9%)	3650 (64.7%)	840 (75.4%)	623 (56.1%)	158 (61.0%)	
Yes	2854 (35.1%)	1991 (35.3%)	274 (24.6%)	488 (43.9%)	101 (39.0%)	
Having at least one daughter						< 0.001
No	5593 (68.8%)	3891 (69.0%)	836 (75.0%)	709 (63.8%)	157 (60.6%)	
Yes	2532 (31.2%)	1750 (31.0%)	278 (25.0%)	402 (36.2%)	102 (39.4%)	
Contraceptive use						0.013
No contraceptive use (No)	4090 (50.3%)	2776 (49.2%)	576 (51.7%)	595 (53.6%)	143 (55.2%)	
Contraceptive use (Yes)	4035 (49.7%)	2865 (50.8%)	538 (48.3%)	516 (46.4%)	116 (44.8%)	
Contraceptive effectiveness category						0.223
LEC	3868 (95.9%)	2748 (95.9%)	519 (96.5%)	494 (95.7%)	107 (92.2%)	
HEC	167 (4.1%)	117 (4.1%)	19 (3.5%)	22 (4.3%)	9 (7.8%)	

*BMI* body mass index, *GIPS* government-affiliated institutions and public sector, *LEC* less effective contraception, *HEC* highly effective contraception

Multivariable logistic regression models were used to examine the association between BMI and contraceptive outcomes. BMI was modeled both as the four-category variable described above and as a continuous variable (per 1 kg/m² increase). The primary outcome was self‑reported contraceptive use prior to the index pregnancy (yes vs. no). The secondary outcome, restricted to contraceptive users, was use of HEC versus LEC. Model 1 was unadjusted. Model 2 was adjusted for hospital center, age, marital status, monthly household income, education level, and occupation. Model 3 was fully adjusted for all covariates in Model 2 plus age at menarche, history of vaginal deliveries, history of cesarean sections, smoking status, and alcohol consumption. Primary and secondary outcomes were analyzed across 10 multiply imputed datasets. Regression coefficients and their standard errors derived from each imputed dataset were pooled using Rubin’s rules to derive pooled odds ratios (ORs), 95% Wald confidence intervals (CIs), and corresponding two-sided *P*‑values.

Two sensitivity analyses were performed to assess the robustness of the findings, with all regression models re-estimated under each scenario: (1) imputation of missing covariate data using the random forest algorithm (R package missForest, version 1.5); (2) complete-case analysis excluding women with missing covariate data.

In the sensitivity analyses, to mitigate potential bias from the Wald normal approximation in subgroups with rare events and small sample sizes, 95% CIs for ORs of secondary outcomes were estimated using the profile likelihood method, and *P*‑values were derived from Type II likelihood ratio tests (the sample size for the primary outcome was sufficient, and the analysis method was consistent with the multiple imputation model). This likelihood‑based framework ensures mathematical coherence between interval estimation and hypothesis testing, and avoids the inconsistencies that may arise from the Wald approximation in underpowered subgroups. Notably, the secondary analysis comparing HEC versus LEC among contraceptive users had limited statistical power, particularly for the obese subgroup, all results for this secondary endpoint are interpreted as exploratory and hypothesis‑generating.

## Results

### Baseline characteristics of the study population

A total of 8,125 women with unintended pregnancies were ultimately included. Table 1 summarizes their baseline characteristics stratified according to BMI category. Significant between-group differences were observed in the below variables (all *P* < 0.05): hospital center, age, marital status, education level, occupation, age at menarche, menstrual regularity, menstrual duration, menstrual flow, dysmenorrhea, history of vaginal deliveries, history of cesarean sections, history of spontaneous abortions, history of induced abortions, smoking status, alcohol consumption, frequency of sexual intercourse, having at least one son, having at least one daughter, and any contraceptive use. No significant differences were detected in monthly household income, menstrual cycle length, history of ectopic pregnancies, or history of molar pregnancies (all *P* > 0.05). Among the subset of women who reported using contraception, no significant difference was observed in the distribution of contraceptive effectiveness categories (*P* > 0.05).

### Association between BMI and contraceptive use

Table 2 presents the association between BMI and reported contraceptive use. In all models, each unit increase in continuous BMI was consistently associated with lower odds of contraceptive use. The fully adjusted OR was 0.98 (95% CI: 0.96–0.99, *P* = 0.003). In categorical analysis, women with overweight had significantly lower odds of contraceptive use in the fully adjusted model, compared to women with normal weight. (OR = 0.85, 95% CI: 0.74–0.98, *P* = 0.024). Similarly, women with obesity also showed significantly lower odds of contraceptive use (OR = 0.75, 95% CI: 0.57–0.98, *P* = 0.035). No significant association was observed among women with underweight (OR = 1.00, 95% CI: 0.87–1.16, *P* = 0.950).

**Table 2:** Associations of BMI with contraceptive use

Variables	Model 1		Model 2		Model 3	
	OR (95% CI)	*P* value	OR (95% CI)	*P* value	OR (95% CI)	*P* value
BMI (Continuous)	0.98 (0.97-1.00)	0.045	0.98 (0.96–0.99)	0.009	0.98 (0.96–0.99)	0.003
BMI (Categorical)						
Normal weight	Ref		Ref		Ref	
Underweight	0.91 (0.80–1.03)	0.128	0.98 (0.86–1.13)	0.828	1.00 (0.87–1.16)	0.950
Overweight	0.84 (0.74–0.96)	0.008	0.87 (0.75–0.99)	0.041	0.85 (0.74–0.98)	0.024
Obese	0.79 (0.61–1.01)	0.059	0.74 (0.57–0.97)	0.032	0.75 (0.57–0.98)	0.035

Model 1 was unadjusted

Model 2 was adjusted for hospital center, age, marital status, education level, monthly household income, and occupation

Model 3 was fully adjusted for all covariates in Model 2 plus age at menarche, history of vaginal deliveries, history of cesarean sections, smoking status, and alcohol consumption

### Association between BMI and contraceptive method category (HEC versus LEC)

Table 3 exhibits the association between BMI and the use of HEC versus LEC among women who reported using contraception. In continuous BMI analysis, no significant association was observed in the fully adjusted model (OR = 1.04, 95% CI: 0.99–1.10, *P* = 0.122). In categorical analyses, no significant associations were detected in women with underweight, overweight, or obesity. (all *P* > 0.05). Given the small proportion of HEC users (4.1% of contraceptive users) and the limited sample size in the obese subgroup, these results should be interpreted as exploratory and hypothesis-generating.

**Table 3:** Associations of BMI with contraceptive method category (HEC versus LEC)

Variables	Model 1		Model 2		Model 3	
	OR (95% CI)	*P* value	OR (95% CI)	*P* value	OR (95% CI)	*P* value
BMI (Continuous)	1.05 (1.00-1.11)	0.042	1.04 (0.98–1.10)	0.162	1.04 (0.99–1.10)	0.122
BMI (Categorical)						
Normal weight	Ref		Ref		Ref	
Underweight	0.86 (0.52–1.41)	0.549	0.87 (0.52–1.44)	0.585	0.85 (0.51–1.41)	0.534
Overweight	1.05 (0.66–1.67)	0.850	0.91 (0.57–1.47)	0.712	0.93 (0.57–1.49)	0.751
Obese	1.98 (0.98-4.00)	0.058	1.81 (0.88–3.76)	0.109	1.89 (0.91–3.92)	0.089

Model 1 was unadjusted

Model 2 was adjusted for hospital center, age, marital status, education level, monthly household income, and occupation

Model 3 was fully adjusted for all covariates in Model 2 plus age at menarche, history of vaginal deliveries, history of cesarean sections, smoking status, and alcohol consumption

### Sensitivity analyses

Baseline characteristics following random forest multiple imputation and complete-case analysis are presented in Supplementary Tables 2 and 3, respectively. Supplementary Table 4 summarizes the association between BMI and reported contraceptive use prior to the index pregnancy under both analytical scenarios. In the random forest multiple imputation model, the fully adjusted OR for BMI as a continuous variable was 0.98 (95% CI: 0.96–0.99, *P* = 0.003), with significant associations persisting in both the overweight and obese groups (OR = 0.85, *P* = 0.024; OR = 0.73, *P* = 0.027, respectively). In the complete-case analysis, the fully adjusted OR for continuous BMI was 0.97 (95% CI: 0.96–0.99, *P* = 0.006). Women with overweight remained at significantly lower odds of contraceptive use (OR = 0.83, *P* = 0.019), while the association was attenuated to non-significance in women with obesity (OR = 0.78, *P* = 0.097).

Regarding the association between BMI and HEC versus LEC use among contraceptive users, results from the random forest multiple imputation dataset and complete-case dataset were consistent with the primary analysis (Supplementary Table 5, Supplementary Table 6). No association was observed between BMI, whether analyzed as a continuous or categorical variable, and the use of HEC or LEC. .

## Discussion

To our knowledge, this is the first large-scale cross-sectional study in China to examine the association between BMI and contraceptive behaviors among women with unintended pregnancy. We observed an independent association between being overweight or obese and a significantly lower probability of contraceptive use, and these findings remained robust across multiple sensitivity analyses. And among contraceptive users, BMI showed no significant association with the HEC versus LEC.

The mechanisms underlying the association between overweight or obese status and the reported contraceptive use prior to the index pregnancy are likely multifactorial. From a psychosocial perspective, women with elevated BMI may experience heightened body image concerns and perceived stigma, potentially leading to avoidance of sexual health discussions and diminished motivation to seek contraceptive counseling [5]. Moreover, women with overweight and obesity also frequently suffer from menstrual dysfunction that impairs ovulation and causes infertility [17]. This biological reality fosters a mistaken belief in their own low fecundity, discouraging contraceptive adoption.

Among women who reported using contraception, there was no statistically significant association between BMI and the HEC versus LEC, whether BMI was analyzed as a continuous variable or as categorical weight groups. Although several theoretical pathways could link higher BMI to use of more effective contraception, including increased clinical attention from healthcare providers, concerns regarding adverse pregnancy outcomes, or non-contraceptive benefits of hormonal methods such as menstrual cycle regulation, our data do not support the presence of a true, reproducible association [18, 19]. This inconsistency is most likely due to the small HEC sample size, which limited the statistical power to detect a true association. Therefore, this observation should be interpreted with caution and requires validation in larger, well-powered prospective studies.

The present study provides a novel two-step analytical framework to examine the association between BMI and contraceptive behavior. We first assessed the association between BMI and any contraceptive use, and then restricted our analysis to contraceptive users to examine whether BMI was associated with the use of contraceptive methods stratified by effectiveness (HEC vs. LEC). While existing literature has focused on the effect of BMI on the efficacy of specific contraceptive methods and the impact of obesity on reproductive and obstetric outcomes [20–24], our study addresses a critical research gap by demonstrating that elevated BMI is associated with lower contraceptive use. These findings reveal a previously underrecognized link between weight status and contraceptive behaviors in China, and provide important evidence for developing targeted contraceptive counseling and reproductive health strategies for women with elevated BMI.

Our study possesses several strengths. First, the multicenter, large-scale design with a sample of 8,125 women provided sufficient statistical power to detect associations. Second, BMI was analyzed as both continuous and categorical variables, yielding consistent results and mitigating subjective cutoff bias. Third, sensitivity analyses using random forest imputation and complete case produced highly consistent findings, which confirms the robustness of our results. Finally, we conducted a DAG-informed approach to comprehensively identify and adjust for confounders, minimizing potential confounding bias.

This study also has several limitations. First, the cross-sectional design precludes does not allow for casual inference, thus future prospective studies are needed to clarify the temporal relationship between BMI and contraceptive behavior. Second, we were unable to adjust for unmeasured confounders such as partner support, which should be included in future research. Third, potential selection bias may exist because all participants were women with unintended pregnancy seeking abortion. The observed associations might partially reflect differential recruitment probability rather than a true difference in contraceptive behavior. Fourth, our primary outcome was a binary measure of any pre-pregnancy contraceptive use, which cannot distinguish between deliberate non-use, inconsistent use, partner barriers, or access barriers. We conducted a secondary analysis among contraceptive users to examine associations between BMI and use of highly effective versus less effective methods for additional insight, but this analysis is strictly exploratory and hypothesis-generating. The overall number of highly effective contraception users was small, particularly in the obese subgroup, which limited the statistical power to detect potential associations. Therefore, no definitive conclusions can be drawn regarding the association between BMI and contraceptive method use, and the findings should be interpreted with caution.

## Conclusions

In conclusion, this large-scale cross-sectional study suggests that overweight and obesity are significantly associated with lower odds of contraceptive use among women seeking abortion for unintended pregnancy in China. These findings highlight the need for integrating weight-specific reproductive health services into family planning care, with the aim of increasing contraceptive use and reducing the burden of unintended pregnancy.

## Supplementary Information

Supplementary Material 1.

## Data Availability

The datasets used and analyzed in this study are available upon reasonable request to the corresponding author.

## Abbreviations

BMI: body mass index
HEC: highly effective contraception
LEC: less effective contraception
OR: Odds ratio
CI: confidence interval
GIPS: government-affiliated institutions and public sector
AIC: Akaike Information Criterion
BIC: Bayesian Information Criterion
IQR: interquartile range
WHO: World Health Organization
