# Supplementary material for: Body mass index and reported contraceptive use prior to the index pregnancy among Chinese women with unintended pregnancy: a multicenter cross-sectional analysis of a prospective cohort
Source: BMC Public Health. 2026 Jul 10;26:2764. doi: 10.1186/s12889-026-28462-2 (PMC13637314; doi:10.1186/s12889-026-28462-2)
Supplement: Supplementary file 1 [file 12889_2026_28462_MOESM1_ESM.docx]

**Supplementary Table**

Supplementary Table 1. Missing data distribution of included study variables.

Supplementary Table 2. Baseline characteristics among women with unintended pregnancies (random forest imputed dataset).

Supplementary Table 3. Baseline characteristics among women with unintended pregnancies (complete-case dataset).

Supplementary Table 4. Associations of BMI with contraceptive use: results from random forest imputation and complete-case analyses.

Supplementary Table 5. Associations between BMI and contraceptive category (HEC vs LEC): random forest imputed dataset.

Supplementary Table 6. Associations between BMI and contraceptive category (HEC vs LEC): complete-case dataset.

Supplementary Figure 1. Directed acyclic graph (DAG) illustrating causal relationships between BMI (exposure) and contraceptive use (outcome). Orange nodes represent confounders included in the minimal sufficient adjustment set; green nodes represent mediators on the causal pathway (not adjusted for); gray nodes represent other variables not required for adjustment. The minimal adjustment set included age, marital status, education level, monthly household income, occupation, hospital center, age at menarche, history of vaginal deliveries, history of cesarean sections, smoking status, and alcohol consumption.

Supplementary Table 1. Missing data distribution of included study variables.

| Variable | Missing Count | Missing Percentage (%) |
| --- | --- | --- |
| Hospital center | 0 | 0 |
| Age | 274 | 3.37 |
| Marital status | 12 | 0.15 |
| Education level | 28 | 0.34 |
| Occupation | 31 | 0.38 |
| Monthly household income | 305 | 3.75 |
| Age at menarche | 12 | 0.15 |
| Menstrual cycle length | 58 | 0.71 |
| Menstrual regularity | 6 | 0.07 |
| Menstrual duration | 68 | 0.84 |
| Menstrual flow | 9 | 0.11 |
| Dysmenorrhea | 24 | 0.3 |
| History of vaginal deliveries | 529 | 6.51 |
| History of Cesarean sections | 683 | 8.41 |
| History of spontaneous abortions | 849 | 10.45 |
| History of induced abortions | 533 | 6.56 |
| History of ectopic pregnancies | 924 | 11.37 |
| History of molar pregnancies | 934 | 11.5 |
| BMI | 0 | 0 |
| Smoking status | 2 | 0.02 |
| Alcohol consumption | 22 | 0.27 |
| Frequency of sexual intercourse | 19 | 0.23 |
| Having at least one son | 619 | 7.62 |
| Having at least one daughter | 653 | 8.04 |
| Contraceptive use | 0 | 0 |
| Contraceptive effectiveness category | 0 | 0 |

Supplementary Table 2. Baseline characteristics among women with unintended pregnancies (random forest imputed dataset).

|  | Overall | Normal weight | Underweight | Overweight | Obese | *P*-value |
| --- | --- | --- | --- | --- | --- | --- |
| N | 8125 | 5641 | 1114 | 1111 | 259 |  |
| Hospital center |  |  |  |  |  | <0.001 |
| 1 | 1696 (20.9%) | 1112 (19.7%) | 239 (21.5%) | 280 (25.2%) | 65 (25.1%) |  |
| 2 | 534 (6.6%) |  | 76 (6.8%) | 82 (7.4%) | 13 (5.0%) |  |
| 3 | 1872 (23.0%) | 1373 (24.3%) | 226 (20.3%) | 224 (20.2%) | 49 (18.9%) |  |
| 4 | 957 (11.8%) | 653 (11.6%) | 147 (13.2%) | 132 (11.9%) | 25 (9.7%) |  |
| 5 | 1377 (16.9%) | 901 (16.0%) | 200 (18.0%) | 219 (19.7%) | 57 (22.0%) |  |
| 6 | 555 (6.8%) | 357 (6.3%) | 77 (6.9%) | 91 (8.2%) | 30 (11.6%) |  |
| 7 | 448 (5.5%) | 381 (6.8%) | 28 (2.5%) | 35 (3.2%) | 4 (1.5%) |  |
| 8 | 72 (0.9%) | 44 (0.8%) | 14 (1.3%) | 11 (1.0%) | 3 (1.2%) |  |
| 9 | 331 (4.1%) | 222 (3.9%) | 77 (6.9%) | 27 (2.4%) | 5 (1.9%) |  |
| 10 | 283 (3.5%) | 235 (4.2%) | 30 (2.7%) | 10 (0.9%) | 8 (3.1%) |  |
| Age, years | 29 (25,32) | 29 (25,32) | 26 (23,30) | 30 (26,33) | 30 (26.5,33) | <0.001 |
| Marital status |  |  |  |  |  | <0.001 |
| Married | 5836 (71.8%) | 4106 (72.8%) | 631 (56.6%) | 885 (79.7%) | 214 (82.6%) |  |
| Others | 2289 (28.2%) | 1535 (27.2%) | 483 (43.4%) | 226 (20.3%) | 45 (17.4%) |  |
| Education level |  |  |  |  |  | <0.001 |
| Primary school education or below | 923 (11.4%) | 607 (10.8%) | 96 (8.6%) | 165 (14.9%) | 55 (21.2%) |  |
| Education prior to bachelor’s degree | 4046 (49.8%) | 2808 (49.8%) | 566 (50.8%) | 548 (49.3%) | 124 (47.9%) |  |
| Bachelor’s degree or higher | 3156 (38.8%) | 2226 (39.5%) | 452 (40.6%) | 398 (35.8%) | 80 (30.9%) |  |
| Monthly household income |  |  |  |  |  | 0.058 |
| <= 4,999 RMB | 3520 (43.3%) | 2429 (43.1%) | 491 (44.1%) | 479 (43.1%) | 121 (46.7%) |  |
| 5,000 ~ 9,999 RMB | 2861 (35.2%) | 1976 (35.0%) | 422 (37.9%) | 384 (34.6%) | 79 (30.5%) |  |
| >=10,000 RMB | 1744 (21.5%) | 1236 (21.9%) | 201 (18.0%) | 248 (22.3%) | 59 (22.8%) |  |
| Occupation |  |  |  |  |  | 0.026 |
| GIPS | 3829 (47.1%) | 2697 (47.8%) | 533 (47.8%) | 495 (44.6%) | 104 (40.2%) |  |
| non-GIPS | 4296 (52.9%) | 2944 (52.2%) | 581 (52.2%) | 616 (55.4%) | 155 (59.8%) |  |
| Age at menarche | 14 (13,14) | 14 (13,14) | 14 (13,15) | 14 (13,14) | 14 (13,14) | 0.003 |
| History of cesarean sections |  |  |  |  |  | <0.001 |
| No | 6097 (75.0%) | 4231 (75.0%) | 947 (85.0%) | 764 (68.8%) | 155 (59.8%) |  |
| Yes | 2028 (25.0%) | 1410 (25.0%) | 167 (15.0%) | 347 (31.2%) | 104 (40.2%) |  |
| History of vaginal deliveries |  |  |  |  |  | <0.001 |
| No | 5250 (64.6%) | 3632 (64.4%) | 787 (70.6%) | 657 (59.1%) | 174 (67.2%) |  |
| Yes | 2875 (35.4%) | 2009 (35.6%) | 327 (29.4%) | 454 (40.9%) | 85 (32.8%) |  |
| Smoking status |  |  |  |  |  | <0.001 |
| Non-smoker | 7902 (97.3%) | 5510 (97.7%) | 1058 (95.0%) | 1079 (97.1%) | 255 (98.5%) |  |
| Smoker | 223 (2.7%) | 131 (2.3%) | 56 (5.0%) | 32 (2.9%) | 4 (1.5%) |  |
| Alcohol consumption |  |  |  |  |  | 0.015 |
| Non-drinker | 7349 (90.4%) | 5088 (90.2%) | 993 (89.1%) | 1032 (92.9%) | 236 (91.1%) |  |
| Drinker | 776 (9.6%) | 553 (9.8%) | 121 (10.9%) | 79 (7.1%) | 23 (8.9%) |  |
| Contraceptive use |  |  |  |  |  | 0.013 |
| No Contraceptive use (No) | 4090 (50.3%) | 2776 (49.2%) | 576 (51.7%) | 595 (53.6%) | 143 (55.2%) |  |
| Contraceptive use (Yes) | 4035 (49.7%) | 2865 (50.8%) | 538 (48.3%) | 516 (46.4%) | 116 (44.8%) |  |
| Contraceptive effectiveness category |  |  |  |  |  | 0.223 |
| LEC | 3868 (95.9%) | 2748 (95.9%) | 519 (96.5%) | 494 (95.7%) | 107 (92.2%) |  |
| HEC | 167 (4.1%) | 117 (4.1%) | 19 (3.5%) | 22 (4.3%) | 9 (7.8%) |  |

*BMI* body mass index, *GIPS* government-affiliated institutions and public sector, *LEC* less effective contraception, *HEC* highly effective contraception

Supplementary Table 3. Baseline characteristics among women with unintended pregnancies (complete-case dataset).

|  | Overall | Normal weight | Underweight | Overweight | Obese | *P*-value |
| --- | --- | --- | --- | --- | --- | --- |
| N | 6663 | 4650 | 891 | 911 | 211 |  |
| Hospital center |  |  |  |  |  | <0.001 |
| 1 | 1364 (20.5%) | 901 (19.4%) | 172 (19.3%) | 240 (26.3%) | 51 (24.2%) |  |
| 2 | 524 (7.9%) | 355 (7.6%) | 74 (8.3%) | 82 (9.0%) | 13 (6.2%) |  |
| 3 | 1746 (26.2%) | 1279 (27.5%) | 210 (23.6%) | 210 (23.1%) | 47 (22.3%) |  |
| 4 | 925 (13.9%) | 629 (13.5%) | 142 (15.9%) | 129 (14.2%) | 25 (11.8%) |  |
| 5 | 505 (7.6%) | 323 (6.9%) | 73 (8.2%) | 83 (9.1%) | 26 (12.3%) |  |
| 6 | 530 (8.0%) | 337 (7.2%) | 76 (8.5%) | 88 (9.7%) | 29 (13.7%) |  |
| 7 | 416 (6.2%) | 352 (7.6%) | 25 (2.8%) | 35 (3.8%) | 4 (1.9%) |  |
| 8 | 69 (1.0%) | 43 (0.9%) | 13 (1.5%) | 10 (1.1%) | 3 (1.4%) |  |
| 9 | 320 (4.8%) | 213 (4.6%) | 77 (8.6%) | 25 (2.7%) | 5 (2.4%) |  |
| 10 | 264 (4.0%) | 218 (4.7%) | 29 (3.3%) | 9 (1.0%) | 8 (3.8%) |  |
| Age, years | 29 (25,32) | 29 (25,32) | 27 (24,31) | 30 (26,33) | 30 (26,33) | <0.001 |
| Marital status |  |  |  |  |  | <0.001 |
| Married | 4919 (73.8%) | 3476 (74.8%) | 528 (59.3%) | 741 (81.3%) | 174 (82.5%) |  |
| Others | 1744 (26.2%) | 1174 (25.2%) | 363 (40.7%) | 170 (18.7%) | 37 (17.5%) |  |
| Education level |  |  |  |  |  | <0.001 |
| Primary school education or below | 780 (11.7%) | 522 (11.2%) | 77 (8.6%) | 135 (14.8%) | 46 (21.8%) |  |
| Education prior to bachelor’s degree | 3387 (50.8%) | 2346 (50.5%) | 479 (53.8%) | 459 (50.4%) | 103 (48.8%) |  |
| Bachelor’s degree or higher | 2496 (37.5%) | 1782 (38.3%) | 335 (37.6%) | 317 (34.8%) | 62 (29.4%) |  |
| Monthly household income |  |  |  |  |  | 0.280 |
| <= 4,999 RMB | 2649 (39.8%) | 1838 (39.5%) | 354 (39.7%) | 365 (40.1%) | 92 (43.6%) |  |
| 5,000 ~ 9,999 RMB | 2467 (37.0%) | 1715 (36.9%) | 355 (39.8%) | 326 (35.8%) | 71 (33.6%) |  |
| >=10,000 RMB | 1547 (23.2%) | 1097 (23.6%) | 182 (20.4%) | 220 (24.1%) | 48 (22.7%) |  |
| Occupation |  |  |  |  |  | 0.073 |
| GIPS | 3231 (48.5%) | 2281 (49.1%) | 443 (49.7%) | 418 (45.9%) | 89 (42.2%) |  |
| non-GIPS | 3432 (51.5%) | 2369 (50.9%) | 448 (50.3%) | 493 (54.1%) | 122 (57.8%) |  |
| Age at menarche | 14 (13,14) | 14 (13,14) | 14 (13,15) | 14 (13,14) | 14 (13,14) | 0.049 |
| History of cesarean sections |  |  |  |  |  | <0.001 |
| No | 4996 (75.0%) | 3475 (74.7%) | 757 (85.0%) | 637 (69.9%) | 127 (60.2%) |  |
| Yes | 1667 (25.0%) | 1175 (25.3%) | 134 (15.0%) | 274 (30.1%) | 84 (39.8%) |  |
| History of vaginal deliveries |  |  |  |  |  | <0.001 |
| No | 4362 (65.5%) | 3035 (65.3%) | 636 (71.4%) | 543 (59.6%) | 148 (70.1%) |  |
| Yes | 2301 (34.5%) | 1615 (34.7%) | 255 (28.6%) | 368 (40.4%) | 63 (29.9%) |  |
| Smoking status |  |  |  |  |  | <0.001 |
| Non-smoker | 6463 (97.0%) | 4532 (97.5%) | 841 (94.4%) | 883 (96.9%) | 207 (98.1%) |  |
| Smoker | 200 (3.0%) | 118 (2.5%) | 50 (5.6%) | 28 (3.1%) | 4 (1.9%) |  |
| Alcohol consumption |  |  |  |  |  | 0.007 |
| Non-drinker | 5967 (89.6%) | 4153 (89.3%) | 781 (87.7%) | 842 (92.4%) | 191 (90.5%) |  |
| Drinker | 696 (10.4%) | 497 (10.7%) | 110 (12.3%) | 69 (7.6%) | 20 (9.5%) |  |
| Contraceptive use |  |  |  |  |  | 0.005 |
| No contraceptive use (No) | 3603 (54.1%) | 2453 (52.8%) | 494 (55.4%) | 533 (58.5%) | 123 (58.3%) |  |
| Contraceptive use (Yes) | 3060 (45.9%) | 2197 (47.2%) | 397 (44.6%) | 378 (41.5%) | 88 (41.7%) |  |
| Contraceptive effectiveness category |  |  |  |  |  | 0.236 |
| LEC | 2920 (95.4%) | 2100 (95.6%) | 379 (95.5%) | 361 (95.5%) | 80 (90.9%) |  |
| HEC | 140 (4.6%) | 97 (4.4%) | 18 (4.5%) | 17 (4.5%) | 8 (9.1%) |  |

*BMI* body mass index, *GIPS* government-affiliated institutions and public sector, *LEC* less effective contraception, *HEC* highly effective contraception

Supplementary Table 4. Associations of BMI with contraceptive use: results from random forest imputation and complete-case analyses.

| Variables | Model 1 |  |  | Model 2 |  |  | Model 3 |  |
| --- | --- | --- | --- | --- | --- | --- | --- | --- |
|  | OR (95% CI) | *P*-value |  | OR (95% CI) | *P*-value |  | OR (95% CI) | *P*-value |
| Random Forest |  |  |  |  |  |  |  |  |
| BMI (Continuous) | 0.98 (0.97-1.00) | 0.045 |  | 0.98 (0.96-0.99) | 0.007 |  | 0.98 (0.96-0.99) | 0.003 |
| BMI (Categorical) |  |  |  |  |  |  |  |  |
| Normal weight | Ref |  |  | Ref |  |  | Ref |  |
| Underweight | 0.91 (0.80-1.03) | 0.128 |  | 0.99 (0.86-1.13) | 0.853 |  | 1.00 (0.87-1.15) | 0.966 |
| Overweight | 0.84 (0.74-0.96) | 0.008 |  | 0.86 (0.75-0.99) | 0.039 |  | 0.85 (0.74-0.98) | 0.024 |
| Obese | 0.79 (0.61-1.01) | 0.059 |  | 0.74 (0.56-0.97) | 0.028 |  | 0.73 (0.56-0.96) | 0.027 |
|  |  |  |  |  |  |  |  |  |
| Complete case |  |  |  |  |  |  |  |  |
| BMI (Continuous) | 0.98 (0.96-1.00) | 0.053 |  | 0.98 (0.96-1.00) | 0.019 |  | 0.97 (0.96-0.99) | 0.006 |
| BMI (Categorical) |  |  |  |  |  |  |  |  |
| Normal weight | Ref |  |  | Ref |  |  | Ref |  |
| Underweight | 0.90 (0.78-1.04) | 0.140 |  | 1.02 (0.88-1.19) | 0.786 |  | 1.05 (0.90-1.22) | 0.563 |
| Overweight | 0.79 (0.69-0.91) | 0.001 |  | 0.85 (0.73-0.99) | 0.033 |  | 0.83 (0.72-0.97) | 0.019 |
| Obese | 0.80 (0.60-1.06) | 0.115 |  | 0.78 (0.58-1.05) | 0.108 |  | 0.78 (0.57-1.05) | 0.097 |

Model 1 was unadjusted.

Model 2 was adjusted for hospital center, age, marital status, education level, monthly household income, and occupation.

Model 3 was fully adjusted for all covariates in Model 2 plus age at menarche, history of vaginal deliveries, history of cesarean sections, smoking status, and alcohol consumption.

Supplementary Table 5. Associations between BMI and contraceptive method category (HEC vs LEC): random forest imputed dataset.

| Variables | Model 1 |  |  | Model 2 |  |  | Model 3 |  |
| --- | --- | --- | --- | --- | --- | --- | --- | --- |
|  | OR (95% CI) | *P*-value |  | OR (95% CI) | *P*-value |  | OR (95% CI) | *P*-value |
| BMI (Continuous) | 1.05 (1.00-1.11) | 0.046 |  | 1.04 (0.98-1.09) | 0.166 |  | 1.04 (0.99-1.10) | 0.135 |
| BMI (Categorical) |  | 0.303 |  |  | 0.394 |  |  | 0.352 |
| Normal weight | Ref |  |  | Ref |  |  | Ref |  |
| Underweight | 0.86 (0.51-1.37) |  |  | 0.87 (0.51-1.41) |  |  | 0.86 (0.50-1.40) |  |
| Overweight | 1.05 (0.64-1.63) |  |  | 0.91 (0.55-1.44) |  |  | 0.92 (0.56-1.46) |  |
| Obese | 1.98 (0.91-3.79) |  |  | 1.84 (0.83-3.62) |  |  | 1.90 (0.86-3.76) |  |

Model 1 was unadjusted.

Model 2 was adjusted for hospital center, age, marital status, education level, monthly household income, and occupation.

Model 3 was fully adjusted for all covariates in Model 2 plus age at menarche, history of vaginal deliveries, history of cesarean sections, smoking status, and alcohol consumption.

Supplementary Table 6. Associations between BMI and contraceptive method category (HEC vs LEC): complete-case dataset.

| Variables | Model 1 |  |  | Model 2 |  |  | Model 3 |  |
| --- | --- | --- | --- | --- | --- | --- | --- | --- |
|  | OR (95% CI) | *P*-value |  | OR (95% CI) | *P*-value |  | OR (95% CI) | *P*-value |
| BMI (Continuous) | 1.04 (0.98-1.10) | 0.219 |  | 1.03 (0.97-1.09) | 0.351 |  | 1.03 (0.97-1.10) | 0.286 |
| BMI (Categorical) |  | 0.338 |  |  | 0.330 |  |  | 0.291 |
| Normal weight | Ref |  |  | Ref |  |  | Ref |  |
| Underweight | 1.03 (0.60-1.68) |  |  | 1.01 (0.58-1.67) |  |  | 0.99 (0.56-1.64) |  |
| Overweight | 1.02 (0.58-1.68) |  |  | 0.91 (0.51-1.52) |  |  | 0.92 (0.52-1.54) |  |
| Obese | 2.16 (0.94-4.35) |  |  | 2.16 (0.92-4.49) |  |  | 2.26 (0.95-4.74) |  |

Model 1 was unadjusted.

Model 2 was adjusted for hospital center, age, marital status, education level, monthly household income, and occupation.

Model 3 was fully adjusted for all covariates in Model 2 plus age at menarche, history of vaginal deliveries, history of cesarean sections, smoking status, and alcohol consumption.
